# Directory for Nurses

**Published:** 1883-02

**Authors:** 


### Directory for Nurses.

In the January number of the Journal, in directing attention to the Buffalo Medical Library Association, we intimated that it was contemplated to establish a "Nurse Registry Bureau," through which the profession may be able to secure at any and all times competent nurses. The necessity of a Directory for Nurses has been recognized for a long time, and we are gratified to witness a movement in the direction of the establishment of a Bureau having this specific object in view. In Philadelphia and Boston such directories have been organized and are in successful operation.

From the first annual report of the "Philadelphia Directory for Nurses" we glean some interesting facts as to the details of management, which, for the information of our readers, we beg to present. The nurse applies for registration and is supplied with the "application blank," which requires a statement of the name, age, address, qualifications, kind of nursing preferred, rates of charge, and both medical and family references. If the replies from the person referred to are satisfactory, the nurse is registered on the payment of a small fee, which has to be paid but once. Detailed inquiries are sent both to the physician and the family after every engagement is terminated, in order to learn more and more of the nurse's qualifications and faults, if there be any of importance. For information to those applying for nurses, a small fee is charged in case of an engagement.

One of the most important results secured through the Directory is the promptness with which calls in great emergencies are met. Cases of accident, sudden illness, etc., often brook no delay. Under our present system, or rather, want of system, it is impossible to obtain the needed assistance without considerable annoyance, and often only with great effort. The amount of time and trouble it saves physicians, who have been compelled to search the city over for a reliable nurse, is, in itself, a positive convenience, as well as a great advantage.

It will be recognized at once that our city needs such an organized agency, the benefits of which will accrue to both the public and the nurses, giving to the former a medium for securing competent and reliable assistance and help in sickness, and to the latter a responsible Directory through which they can secure employment.

To the profession the Directory will prove a source of convenience and aid, and inasmuch as in its organization it will depend upon the encouragement and endorsement of the physicians of the city, we trust that this brief statement of its objects and purposes will be carefully considered. We will place before the profession all information in regard to its organization as soon as it is perfected.

